# Near‐Fatal Arrhythmia From Severe Mitral Annular Disjunction

**DOI:** 10.1155/cric/2912144

**Published:** 2026-06-26

**Authors:** Peter-Jon Williams, Mark Schwade, Ryan F. Bloomquist, Robert Sorrentino, Deya A. Alkhatib, Christine Mills

**Affiliations:** ^1^ School of Medicine, Medical College of Georgia, Augusta University, Augusta, Georgia, USA, augusta.edu; ^2^ Department of Cardiology, Medical College of Georgia, Augusta University, Augusta, Georgia, USA, augusta.edu; ^3^ Department of Ophthalmology, Medical College of Georgia, Augusta University, Augusta, Georgia, USA, augusta.edu

## Abstract

Mitral annular disjunction (MAD) is increasingly recognized as a structural abnormality associated with ventricular arrhythmias and sudden cardiac arrest, particularly in the setting of bileaflet mitral valve prolapse (MVP). A 43‐year‐old man with a history of migraines presented following a witnessed out‐of‐hospital cardiac arrest and was successfully resuscitated with a single AED shock. Transthoracic echocardiography showed mitral regurgitation with bileaflet prolapse and suspected MAD. An implantable cardioverter‐defibrillator was placed for secondary prevention, and the patient was discharged in stable condition with outpatient surgical follow‐up where robotic‐assisted mitral valve repair was successfully performed. Advanced imaging plays a pivotal role in diagnosis and risk stratification when MAD presents as sudden cardiac arrest.

**Take Home Message**

Timely recognition of MAD in patients presenting with syncope or sudden cardiac arrest is essential as it may directly influence both implantable cardioverter‐defibrillator (ICD) placement for secondary prevention and surgical planning.

## 1. History of Presentation

A 43‐year‐old man suffered out‐of‐hospital cardiac arrest while seated at work. Coworkers initiated chest compressions before an automated external defibrillator delivered a therapeutic shock. Emergency medical services arrived thereafter and transported the patient to the hospital for further evaluation. On arrival at the hospital, neurologic exam was intact, and cardiac exam was significant for a 2/6 systolic murmur found in the fifth intercostal space and radiating to the axilla.

## 2. Medical History

The patient did not have a significant past medical history. He was athletic and stated he often ran long distances. He denied prior episodes of syncope.

## 3. Differential Diagnosis

Differential diagnosis of arrest included ventricular tachycardia and ventricular fibrillation, the cause of which could include structural or ischemic heart disease. Other causes could include long QT syndrome or Brugada syndrome.

## 4. Investigations

**Figure 1:**
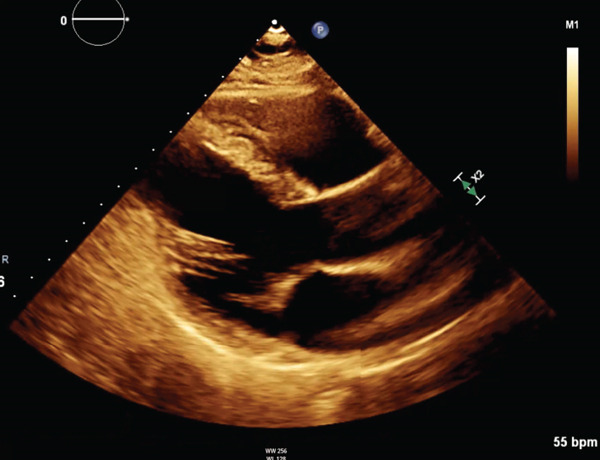
Markedly myxomatous anterior leaflet with moderate prolapse and systolic curling of the posterior annulus with suggestion of mitral annular disjunction.

**Figure 2:**
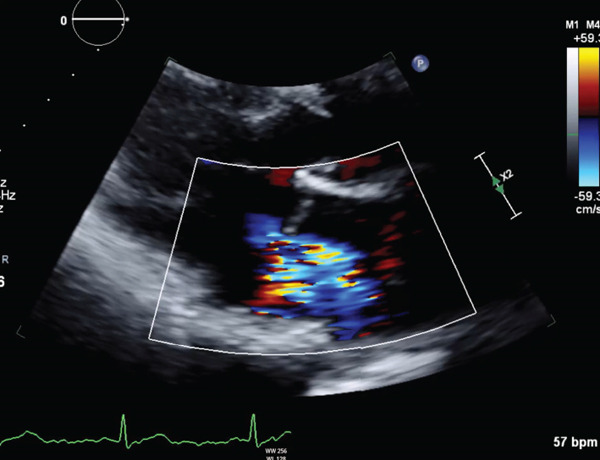
Color Doppler imaging demonstrates significant mitral regurgitation indicated by color flow.

Laboratory testing was significant for lactic acid of 4.1 mmol/L, bicarbonate of 19 mmol/L, AST of 144 U/L, and ALT of 129 U/L. Troponins were mildly elevated at 0.063 ng/mL, downtrending to 0.042 ng/mL. Electrocardiogram demonstrated normal sinus rhythm with incomplete right bundle branch block and T wave inversions in Lead III. Transthoracic echocardiography showed normal left ventricular size, wall thickness, and systolic function, with an estimated ejection fraction of 51%–55%. Notably, bileaflet MVP was observed including a markedly myxomatous anterior leaflet with moderate prolapse and systolic curling of the posterior annulus with suggestion of mitral annular disjunction (Figure 1). Color Doppler imaging revealed significant mitral regurgitation (Figure 2). Coronary angiography was performed which revealed only mild luminal irregularities.

**Figure 3:**
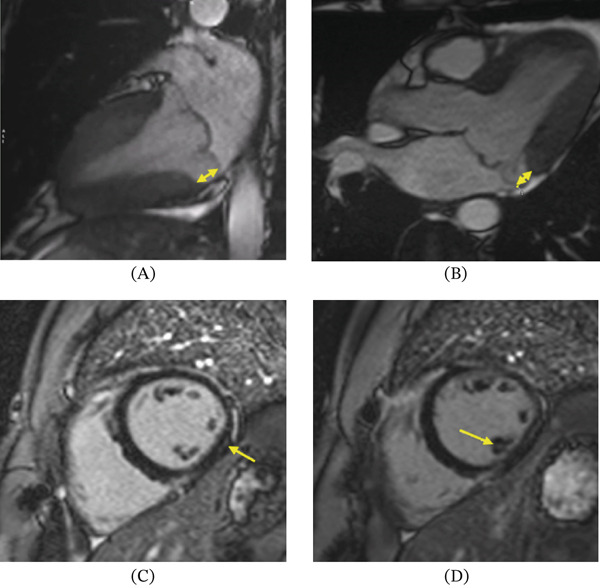
(A) and (B) demonstrate myxomatous mitral valve with significant bileaflet prolapse as well as mitral annular disjunction of 11 mm. Image (C) shows focal midmyocardial LGE of the basal inferolateral segment, whereas (D) demonstrates LGE in the tip of the posteromedial papillary muscle in a nonischemic pattern.

**Figure 4:**
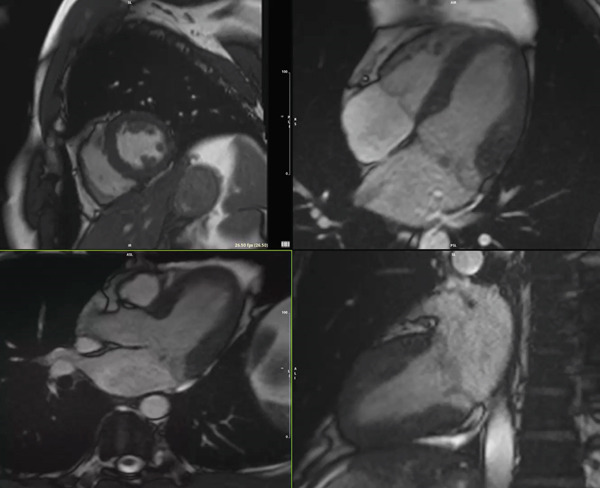
Curling of the basal inferolateral segment noted, with exaggerated basal‐to‐mid wall thickness ratio demonstrated across synchronized planes.

To further characterize valvular and myocardial structure, cardiac magnetic resonance (CMR) imaging was performed. CMR confirmed a myxomatous mitral valve with significant bileaflet prolapse and a measured mitral annular disjunction of 11 mm (Figure 3 A,B). There was severe primary mitral regurgitation with a regurgitant fraction of 53% and a regurgitant volume of 42 mL/beat. The left ventricle was mildly enlarged with a left ventricular end‐diastolic volume index of 108 mL/m^2^ and preserved systolic function (LVEF 55%). Additionally, curling of the basal inferolateral segment was noted with an exaggerated basal‐to‐mid wall thickness ratio (Figure 4). Late gadolinium enhancement (LGE) was observed in the basal inferolateral segment and at the tip of the posteromedial papillary muscle in a nonischemic midmyocardial pattern consistent with focal fibrosis related to underlying MAD (Figure 3 C,D).

## 5. Management

Given the patient′s cardiac arrest in the setting of MAD, bileaflet MVP, and myocardial fibrosis, the electrophysiology service proceeded with placement of an ICD for secondary prevention. The patient remained hemodynamically stable throughout hospitalization and was discharged.

## 6. Outcome and Follow‐Up

The patient had close follow‐up in the cardiology device clinic and plans for outpatient surgical evaluation for mitral valve repair. He ultimately underwent robotic‐assisted mitral valve repair at a different quarternary care center, which we have reviewed the records of and provided details of operative management as follows. Patient underwent triangular resection of the P2/P1 leaflet of the mitral valve with subsequent reapproximation, and two pairs of neochords were placed and fixed from the papillary muscles to the A2 scallop. An annuloplasty ring of 36 mm was used, and while the edges of the band were being sutured to the trigones and posterior annulus, deep bites were taken to include a portion of the posterior leaflet to relocate the annulus to the appropriate location to eliminate the annular disjunction. Given focal fibrosis of the papillary muscle seen on cardiac MRI, cryotherapy device was used to ablate and isolate the papillary muscle to reduce risk of recurrent ventricular tachycardia. A postoperative TTE was obtained on post‐op Day 2 and showed only trace mitral regurgitation with a mean gradient of 2 mmHg with no residual mitral annular dysjunction. There was low normal systolic function with a left ventricular ejection fraction of 50%.

## 7. Discussion

Mitral annular disjunction is a structural abnormality characterized by a separation between the mitral valve annulus and the basal left ventricular myocardium. First described in the 1980s by Hutchins et al. in autopsy studies of myxomatous mitral valves, MAD was initially considered an anatomic variant linked to excessive leaflet motion and valve degeneration [1]. This disjunction impairs the normal systolic contraction of the annulus, which contributes to incomplete leaflet coaptation and functional mitral regurgitation even in the absence of overt leaflet pathology. The annular separation is often dynamic and may be exacerbated by increased left ventricular contractility or afterload. Although MAD can occur in both sexes, it has been found to be more prevalent among women with MVP. Men, like the patient described here, are more frequently referred for surgical intervention due to progression to severe MR [2]. In recent years, MAD has been implicated in arrhythmogenesis largely due to its association with myocardial fibrosis and mechanical stretch at the inferobasal left ventricular wall and papillary muscle apparatus. A landmark study by Dejgaard et al. [3] demonstrated that MAD can occur independently of MVP and is itself an arrhythmogenic entity. It is associated with ventricular arrhythmias and even sudden cardiac arrest in the absence of significant mitral leaflet abnormalities or regurgitation severity by echocardiography.

Diagnosis of MAD requires high‐resolution imaging as TTE often fails to capture the subtle systolic curling or paradoxical annular motion that characterizes the disorder. In this case, TTE identified bileaflet mitral valve prolapse with significant MR but underestimated the extent of structural abnormality. CMR proved essential by demonstrating an 11‐mm annular disjunction and revealing focal LGE in the basal inferolateral segment and posteromedial papillary muscle. These findings were consistent with localized fibrosis in a nonischemic pattern and likely reflect the mechanical and electrical consequences of annular instability. MRI remains the gold standard for quantifying annular disjunction and detecting associated myocardial fibrosis as supported by a systematic review demonstrating significant variability in echocardiographic detection and emphasizing the superior spatial resolution and tissue characterization provided by MRI [4].

From a clinical standpoint, MAD is increasingly recognized as a risk factor for ventricular arrhythmias and sudden cardiac death, especially in the context of bileaflet MVP. Although our patient had no known arrhythmic history prior to presentation, his out‐of‐hospital cardiac arrest in the setting of MAD and LGE strongly supports a diagnosis of arrhythmogenic MAD. Recent literature further underscores MAD as a distinct structural and electrical substrate for arrhythmias, with manifestations in young patients with MVP and myocardial fibrosis. Its recognition is critical as many individuals remain asymptomatic until a sentinel event occurs, and disjunction may be overlooked without high‐resolution imaging. Prevalence estimates vary widely depending on imaging modality, patient population, and diagnostic thresholds. In the general population, MAD has been reported in 7.2%–8.7% of individuals and appears more common in young women. Among patients with MVP, MAD prevalence ranges from 16% to 92% in postmortem studies, with more recent imaging studies suggesting a prevalence of approximately 30% and translating to an estimated 1–2 million individuals in the United States [5]. These figures emphasize the close relationship between MAD and MVP, though the prevalence of clinically significant arrhythmogenic MAD remains uncertain.

There may also be a subset of MAD patients with benign features without LGE on CMR. This subtype is described by Sonaglioni et al. [6] as having a narrow A‐P thoracic diameter. The anterior chest wall extrinsically compresses upon the cardiac chambers, leading to smaller chamber sizes and inducing MAD. They hypothesize that this MAD subtype may carry a lesser arrhythmogenic burden and describe a case with MAD of 11 mm and without any LGE on CMR in an asymptomatic patient [7]. This highlights the important role that CMR plays in risk stratifying patients with MAD and the importance of identifying any myocardial fibrosis that could lead to arrhythmia, as an asymptomatic patient may not benefit from mitral valve repair or defibrillator placement.

Therapeutic strategies for MAD remain evolving. Although mitral valve repair with annuloplasty can effectively stabilize the annular‐ventricular junction and reduce mitral regurgitation, both repair and replacement have been shown to correct the disjunction by anchoring the annulus to the left ventricular myocardium. However, despite observed reductions in arrhythmic burden following surgery, it remains unclear whether surgical intervention definitively lowers the long‐term risk of malignant arrhythmias or sudden cardiac death in this population [8]. In our case, the surgeon decided to perform cryoablation to the posteromedial papillary muscle. This decision was likely guided by the cardiac MRI we obtained which showed LGE at the tip of this papillary muscle. This may represent the foci of the patient′s arrhythmia as MAD can cause inflammation and stress on the papillary muscle, which can lead to fibrosed myocardial tissue. This is a novel intervention as although mitral valve repair may eliminate ongoing inflammation and myocardial injury, any residual scar may lead to recurrent ventricular tachycardia. By performing intraoperative ablation, we may more successfully reduce future arrhythmic burden.

AICD placement remains the cornerstone of secondary prevention in survivors of sudden cardiac arrest as was performed in our patient. In a cohort of patients with MAD and ICDs, 37% received appropriate ICD therapies most commonly for polymorphic ventricular tachycardia or ventricular fibrillation, which underscores the high arrhythmic burden in this population [9]. Notably, those with a history of sudden cardiac arrest and lower left ventricular ejection fraction were significantly more likely to benefit from ICD intervention. Given this structural and arrhythmic risk, ongoing rhythm monitoring and shared decision‐making regarding surgical timing are essential.

## 8. Conclusion

Mitral annular disjunction is a structural abnormality increasingly recognized as both a mechanical contributor to mitral regurgitation and an independent substrate for malignant ventricular arrhythmias. Although frequently associated with mitral valve prolapse, MAD can occur independently and may be underdiagnosed due to the limited sensitivity of transthoracic echocardiography. This case adds to the growing literature on MAD by illustrating out‐of‐hospital cardiac arrest in a relatively young patient with bileaflet MVP, significant mitral regurgitation, and MAD. It emphasizes the critical importance of integrating TTE and CMR imaging to fully characterize the mitral apparatus and surrounding myocardium. MRI in particular offers superior tissue characterization and allows for both accurate quantification of annular disjunction and detection of LGE, which are hallmarks of arrhythmogenic risk. Although surgical correction may restore annular stability, its impact on long‐term arrhythmic outcomes remains uncertain.

## Funding

No funding was received for this manuscript.

## Disclosure

All authors contributed meaningfully to this work and meet criteria for authorship.

## Consent

Consent was obtained to publish this deidentified case.

## Conflicts of Interest

The authors declare no conflicts of interest.

## Data Availability

Data sharing is not applicable to this article as no datasets were generated or analyzed during the current study.
